# Comparing HIV prevalence estimates from prevention of mother-to-child HIV transmission programme and the antenatal HIV surveillance in Addis Ababa

**DOI:** 10.1186/1471-2458-12-1113

**Published:** 2012-12-26

**Authors:** Alemnesh H Mirkuzie, Mitike Molla Sisay, Sven Gudmund Hinderaker, Karen Marie Moland, Odd Mørkve

**Affiliations:** 1 Centre for International Health, University of Bergen, Overlege Danielsens Hus, Årstav. 21, Bergen, 5020, Norway; 2 Department of Nursing and Midwifery, College of Medical and Health Sciences, Hawassa University, p.o.box 1560, Awassa, Ethiopia; 3 Behavioural Health Sciences Unit, School of Public Health, Collage of Health Sciences, Addis Ababa University, p.o.box 9086, Addis Ababa, Ethiopia

**Keywords:** Addis Ababa, ANC surveillance, HIV prevalence, PMTCT programme reports

## Abstract

**Background:**

In the absence of reliable data, antenatal HIV surveillance has been used to monitor the HIV epidemic since the late 1980s. Currently, routine data from Prevention of Mother-to-child HIV transmission (PMTCT) programmes are increasingly available. Evaluating whether the PMTCT programme reports provide comparable HIV prevalence estimates with the antenatal surveillance reports is important. In this study, we compared HIV prevalence estimates from routine PMTCT programme and antenatal surveillance in Addis Ababa with the aim to come up with evidence based recommendation.

**Methods:**

Summary data were collected from PMTCT programmes and antenatal surveillance reports within the catchment of Addis Ababa. The PMTCT programme data were obtained from routine monthly reports from 2004 to 2009 and from published antenatal HIV surveillance reports from 2003 to 2009. Data were analysed using descriptive statistics.

**Results:**

In Addis Ababa, PMTCT sites had increased from six in 2004 to 54 in 2009. The site expansion was accompanied by an increased number of women testing. There were marked increases in the rate of HIV testing following the introduction of routine opt-out HIV testing approach. Paralleling these increases, the HIV prevalence showed a steady decline from 10.0% in 2004 to 4.5% in 2009. There were five antenatal surveillance sites from 2003 to 2007 in Addis Ababa and they increased to seven by 2009. Four rounds of surveillance data from five sites showed a declining trend in HIV prevalence over the years. The overall antenatal surveillance data also showed that the HIV prevalence among antenatal attendees had declined from 12.4% in 2003 to 5.5% in 2009. The HIV prevalence estimates from PMTCT programme were 6.2% and 4.5% and from antenatal surveillance 6.1 and 5.5% in 2008 and 2009 respectively.

**Conclusions:**

There were consistent HIV prevalence estimates from PMTCT programme and from antenatal surveillance reports. Both data sources showed a marked decline in HIV prevalence among antenatal care attendees in Addis Ababa. This study concludes that the routine data from the PMTCT programmes in Addis Ababa provides comparable HIV prevalence estimates with antenatal HIV surveillance data and could be used for monitoring trends.

## Background

Antenatal HIV surveillance is widely used to monitor trends in HIV prevalence and to model incidence rates [1-6]. It has also been used to direct efforts towards HIV/AIDS prevention and control in the absence of reliable data for HIV/AIDS programme monitoring and evaluation. For antenatal surveillance, blood samples collected from pregnant women for routine syphilis tests are used. Since the samples are unlinked and anonymous, participation bias is unlikely [6,7].

Despite its popularity, there are ethical and methodological concerns about antenatal HIV surveillance which remain unsolved [6,7]. From an ethical point of view, the antenatal surveillance does not offer direct individual level benefits to the women since the test is anonymous and unlinked. The major methodological issue includes its reliance on samples collected from a few selected sites for a certain period in a year, lack of representation of private health facilities, sites are often urban biased and the sample sizes are often arguable. Not being part of routine activity, costs associated with antenatal surveillance system are additional concerns. Prabhu et al. have done an economic evaluation in Zanzibar to estimate incremental costs of antenatal surveillance using routine Prevention of mother-to-child HIV transmission (PMTCT) programme data and reported that routine PMTCT report provides considerable cost saving compared to antenatal surveillance [8].

However, although no additional costs are incurred in routine PMTCT programme reports, their validity is affected by low antenatal care coverage, low rate of HIV test acceptance and lack of standardized recording and reporting system.

In Ethiopia, following the rapid scaling up of PMTCT programmes, large proportions of pregnant women have been reached [9,10]. At the national level the number of health facilities offering PMTCT services in 2009 was over 10 times higher than the number of antenatal surveillance sites i.e. 1 352 vs 114 respectively. There are large variations in coverage of antenatal care and uptake of HIV testing in PMTCT programmes across the regions in Ethiopia. The capital Addis Ababa, has the highest antenatal coverage as well as the highest uptake of antenatal HIV testing. In 2012 Ethiopian Demographic and Health Survey reports, 94% pregnant women in Addis Ababa receive antenatal care from skilled health care providers and 84% receive skilled birth care [11]. Moreover, about 85% antenatal attendees receive HIV testing [10]. In 2009, there were 54 PMTCT sites and only seven antenatal HIV surveillance sites across the city. All the seven surveillance sites also provided PMTCT programmes.

Therefore, this study compared HIV prevalence estimates from routine PMTCT programmes and antenatal surveillance in Addis Ababa using summary reports. The study used Addis Ababa as a case for preliminary analysis whether routine PMTCT programme reports have a potential to monitor HIV prevalence trends and to come up with evidence based recommendations.

## Methods

This retrospective study used PMTCT programme and antenatal HIV surveillance reports for the whole catchment of Addis Ababa. Addis Ababa is administratively divided into 10 sub-cities. Under each sub-city there are public and private health facilities reporting their routine monthly activities including PMTCT. Five public health centres from 2003 to 2007 and seven in 2009 have been dual sites for regular antenatal surveillance and PMTCT programme. At the sub-city level, monthly PMTCT reports from all health facilities are compiled together and reported to the City Council Health Bureau. This study compiled routine monthly PMTCT reports from all the sub-cities and the antenatal surveillance reports from published source.

### PMTCT programme reports

Free PMTCT programme was launched in Addis Ababa in 2004 in six selected public health centres located in three sub-cities. By 2005, the programme was expanded to all the ten sub-cities but the expansion took place at different times. The programme has continued to scale up and from 2007 it has expanded into private health facilities. Currently, almost all health facilities providing maternity care services in the city also provide PMTCT services.

In early 2008, the HIV testing approach was shifted from opt-in (voluntary testing) to opt-out (routine testing) [10]. In the programme HIV counselling and testing is offered as part of antenatal care for all pregnant women. PMTCT providers in the antenatal clinics perform a rapid HIV testing from a finger prick blood sample. There is re-testing of HIV positive samples for verification before informing the test result to the woman. In cases of inconsistencies between first and second tests a third tie breaker test will be used. Following the testing, the women’s HIV test result and other PMTCT information are recorded in PMTCT logbooks at the facilities. End of each month, PMTCT service providers at the health facilities record summary PMTCT reports on standard PMTCT format distributed by the sub-cities. This format include summary data on the number of first time antenatal attendees, number of revisit antenatal attendees, number of women who received pre-test HIV counselling, number of women who received HIV testing, number of women who received post-test counselling and number of women tested positive [10]. These summary data were all analysed in this study.

In this study due to the lack of individual level and site specific data in the sub-cities, the HIV prevalence estimates were calculated from summary monthly reports. The monthly PMTCT reports from 2004 to 2009 were collected from all the 10 sub-cities. For the purpose of data validation, reports were also collected from Addis Ababa City Council Health Bureau and Intra-Health (an NGO working on PMTCT). During the data collection, 3 months reports were missing in one of the sub-city (Lideta) but we collected them from the service outlets. Of the collected monthly reports, 6.5% had some incomplete information.

### Antenatal surveillance reports

Summary antenatal HIV surveillance data was obtained from the 2007 and 2009 rounds antenatal HIV surveillance report published by the Federal Ministry of Health of Ethiopia and Ethiopian Health and Nutrition Research and from AIDS in Ethiopia fifth and sixth reports [12-15]. Ethiopia uses antenatal surveillance for monitoring HIV prevalence trends and modelling incidence across the county. Surveillance sites are selected based on set of criteria including suitability for antenatal care, accessible functional laboratory, adequate client volume, sustainable supply for syphilis screening and routine blood drawing services for syphilis/haemoglobin testing. Three to four hundred left over blood samples for routine syphilis test are used. The sample collection period is 12 weeks from urban sites whereas for 20 weeks from rural sites. The samples are anonymous and unlinked collected consecutively from 15–49 years old antenatal attendees during the surveillance period. After collection, specimens are transported to regional testing laboratories and screened using Vironostika HIV antigen/antibody Enzyme Immunosorbent Assay (EIA). All HIV positive specimens are re-tested using Murex HIV antigen/antibody EIA [12].

The first antenatal surveillance site was established in 1989 in one public health centre [16]. In 1989 the HIV prevalence among antenatal attendees was 4.6% which has peaked to 21.2% in 1995 and followed by steady decline since then [12]. From 1997 more sites were added but limited to public health facilities. Since 2003, there were five health centres namely Akaki, Gulele, Higher 23, Kazanchis and Teklehymanot conducting regular antenatal surveillance while Kolfe and Kotebe health centres were included from 2009. In this study, for the trend analysis the five health centres which had complete reports from 2003 to 2009 (four consecutive) rounds were used.

### Ethical considerations and data analysis

Ethical clearance was obtained from Addis Ababa City Council Health Bureau and from the Regional committee for Medical Research Ethics in Western Norway. Study permits were obtained from respective sub-cities health bureaus and health facilities. Data were double entered and checked for consistencies before analysis. The monthly PMTCT reports were entered in Microsoft Excel spread sheet and analyzed using Pivotal table and Pivotal chart report. Data analyses were done using descriptive statistics.

## Results

### The PMTCT reports

**Figure 1:**
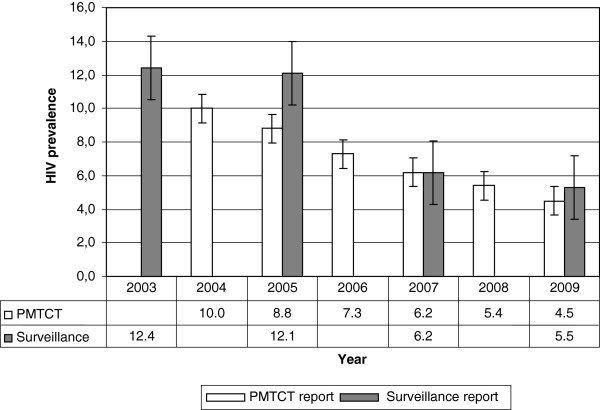
Comparing HIV prevalence estimates from PMTCT and antenatal surveillance reports from 2003–2009 in Addis Ababa.

In Addis Ababa, as the number of facilities providing PMTCT services increased from six in 2004 to 54 in 2009, the number of women receiving HIV testing also showed a year by year increase (Table 1). The reports in 2009 compiled data for 9 months, and gave lower number of women tested in 2009 than in 2008. From 2004 up to 2007 antenatal HIV counselling and testing was offered in voluntary basis (opt-in approach), and the antenatal attendees tested for HIV were 66% in 2004, 33% in 2005, 40% in 2006 and 52% in 2007. Following the introduction of routine opt-out testing approach, 75% of the antennal attendees in 2008 and 84% in 2009 were tested for HIV (Table 1). Across the year the HIV prevalence showed steady decline from 10.0% in 2004 to 4.5% in 2009 (Figure 1).

**Table 1:** Number of antenatal care attendees in health facilities providing PMTCT services, those tested for HIV and the HIV prevalence from 2004 to 2009 in Addis Ababa

**Year**	**Number of antenatal attendee in PMTCT facilities**	**Number of women HIV tested in PMTCT programme**	**HIV prevalence**
**2004**	6208	4097	10.0
**2005**	46272	15131	8.8
**2006**	58510	23170	7.3
**2007**	54502	28450	6.2
**2008**	47591	35741	5.4
**2009**	34585	29397	4.5

### Antenatal surveillance reports

In Addis Ababa, five health centres from 2003–2007 and seven in 2009 had conducted regular antenatal HIV surveillance. In total 1 452 pregnant women in 2003, 1 939 in 2005, 1 517 in 2007 and 2 754 were tested for antenatal HIV surveillance (Table 2). Data collected from the five health centres which participated in four consecutive rounds of surveillance showed declining trends in HIV prevalence (Table 2). The two antenatal HIV surveillance sites added in 2009 i.e. Kolfe and Kotebe reported a lower HIV prevalence than those who had been reporting for years. The overall HIV prevalence had declined across the years from 12.4% in 2003 to 12.1% in 2005 then 6.2% in 2007 and reached 5.5% in 2009 (Figure 1).

**Table 2:** Antenatal HIV surveillance reports stratified by health centres from 2003 - 2009

**Health centre**	**Total number of women tested for HIV (HIV prevalence in%)**			
	**2003**	**2005**	**2007**	**2009**
Akaki	304 (10.9)	428 (9.1)	309 (7.8)	420 (7.4)
Gulele	306 (12.4)	393 (13.0)	310 (6.1)	310 (8.7)
Higher 23	306 (11.8)	444 (10.1)	341 (5.2)	333 (5.4)
Kazanchis	284 (11.6)	341 (16.7)	283 (5.7)	497 (4.4)
Teklehymanot	252 (15.1)	333 (11.7)	274 (6.2)	304 (6.9)
Kolfe				497 (2.2)
Kotebe				393 (3.8)

## Discussion

In this study, we compared HIV prevalence estimates from routine PMTCT programme and antenatal HIV surveillance in Addis Ababa with the intention to come up with evidence based recommendations on the potential of PMTCT programme data for antenatal HIV surveillance. The proportion of women testing in PMTCT programme increased markedly, particularly following the introduction of the opt-out HIV testing approach. According to the summary reports, the HIV prevalence among pregnant women in Addis Ababa had shown steady decline. Four years trend data from the five health centres that conducted regular antenatal HIV surveillance also confirmed the decline. The HIV prevalence estimates obtained from the PMTCT programmes and the antenatal surveillance showed consistencies. In conclusion, the routine PMTCT programme report in Addis Ababa can provide comparable estimate with that of antenatal HIV surveillance report and has potential to monitor HIV prevalence trends.

Our findings showed that the proportion of women testing in PMTCT programme increased markedly especially following the introduction of the opt-out HIV testing approach. Consistent with our findings, several studies from resource poor settings including Ethiopia have reported dramatic increases in the proportions of women testing in PMTCT programmes following the WHO policy statement on routine opt-out HIV testing approach in 2004 [10,17-19]. The data obtained from the different sources reported that the HIV prevalence among pregnant women in Addis Ababa had shown steady decline. This is in agreement with a paper of Mirkuzie et al., where a declining HIV prevalence among antenatal care attendees in Addis Ababa was documented [10]. In the 2011 UNAIDS reports, the HIV epidemic in Ethiopia among 15–24 years old pregnant women show 82% decline in 2010 compared to 2001 [20].

In our study, the HIV prevalence estimates from the PMTCT programmes and from the antenatal HIV surveillance showed high consistency in 2008 and 2009, where the number of women testing in PMTCT programme increased following the introduction of opt-out testing approach. Similar reports were documented in Cameroon with 69% of women testing in PMTCT programme, comparable HIV prevalence estimates were reported i.e. 7.8% from PMTCT programme and 7.3% from antenatal surveillance. Also, in Botswana when HIV testing in PMTCT programme was >95% between 2005 and 2007, the HIV prevalence estimates from PMTCT programme and antenatal surveillance were similar [21]. In India with 68% rate of HIV testing in PMTCT programme no statistically significant difference was observed between the two estimates [2]. In our study, when the rate of HIV testing in PMTCT programme was lowest; 33% in 2005 there appeared to be disparities between the two estimates, 8.8% in the PMTCT programme and 12.1% in the antenatal surveillance (Figure 1). Using the 2002 and 2003 PMTCT reports in Uganda, Mpairwe and colleagues also reported higher HIV prevalence from PMTCT programme than antenatal surveillance with less than 70% of the antenatal attendees testing for HIV in the PMTCT programme but no differences with 70% or more of the antenatal attendees testing [22].

Marsh et al. have analysed papers from seven sub-Sahara African countries; when the rate of HIV testing is low the possibility of participation bias in PMTCT programmes is high due to perception of risks and differences in demographic characteristics. Participation bias can also happen if the PMTCT programme is newly established as high risk cases are more likely to take advantage of the HIV testing [21]. In general, participation bias is less likely where routine opt-out HIV approach is properly implemented. Routine antenatal HIV testing has been implemented in Addis Ababa since early 2008 and accompanied by significant improvement in the rate of HIV testing in PMTCT programmes, and in other resource poor settings [10,17,18]. Marsh and colleagues also assessed the comparability of the two estimates; papers from Cameroon and Botswana asserted the comparability of the two estimates and suggesting its potential utility for HIV monitoring. In contrast, although the overall HIV prevalence estimates appear to be similar in Kenya, Uganda and Zimbabwe there are large differences at clinic level and are discouraging the replacement of PMTCT programme estimate for the antenatal surveillance estimate [21]. Consistent with the latter observations, a report from 43 dual PMTCT and surveillance sites in Ethiopia showed comparable overall HIV prevalence estimates but large differences at clinic level [12].

Although all these studies acknowledged the potentials of the PMTCT programme data for antenatal HIV surveillance purposes, they all agreed that currently its quality is not good enough to be used for surveillance. The major concerns are lack of uniform registers, reporting formats and reporting date and missing or inconsistent training among those who are doing the routine reporting. Issues related to HIV testing include lack of standard testing algorithm, unreliable supply of test kits and other logistics, variable training among professionals who are doing the testing and lack of quality control system [12,21]. The lack of international consensus on how to make routine PMTCT reports suitable for antenatal surveillance is another concern [21].

As the HIV epidemic is maturing and is taking a chronic course, it is imperative to revisit existing strategies to maximize efficiency of the control programme and to direct resources where it is most needed. Larson and colleagues in their paper highlighted the need for strategic responses, including integration of AIDS intervention with other relevant health programmes, and the need for regular systematic programme monitoring and evaluation [23]. Currently, the Global Health Initiative (GHI) is investing on health system strengthening for optimal health gain and is supporting the Ethiopian government [24]. Being one of the building blocks of the health system, improving monitoring and evaluation of programme activities are emphasized.

For optimal monitoring and evaluation, Health Management Information System (HMIS) has been implemented in Addis Ababa for routine programme reporting [25]. The HMIS use standard computerized registers and reporting system and it can be an asset for standardizing the PMTCT reports. Even before the scaling up of the HMIS across the city, the quality of the PMTCT reports seem to be good; over 90% the reports from the sub-cities were complete. Moreover, standard antenatal HIV testing algorithm recommended in the 2007 Ethiopian PMTCT guidelines has been used in Addis Ababa, and could help to minimize potential inconsistencies at the level of sample collection and processing [26].

In line with these arguments, Addis Ababa seems to quality to use routine PMTCT programme reports for surveillance purpose and can be a pilot site for Ethiopia. Nonetheless, continuing the regular antenatal HIV surveillance in parallel for some time could help to validate the quality of PMTCT programme data until it gets perfected. Meanwhile, issues related to supply of test kits and logistics, particularly the need for continuous in-service training to minimize gaps due to staff turnover need to be emphasized.

Use of summary reports and the lack of cost effectiveness analysis would be some major limitations of the study. However, to ensure quality, the PMTCT reports obtained from the sub-cities were validated with reports from the City Council Health Bureau and from Intra-Health and they were consistent. For ensuring data completeness, missing monthly reports were collected from service outlets. The study could have benefited from cost effectiveness analysis to show how much saving the health system can make when antenatal surveillance is phased out and investment is diverted for generating quality PMTCT programme reports. We used summary reports from PMTCT reporting formats aggregated at the sub-city level and summarized HIV prevalence reports, and hence we lack individual level and clinic based data to assess differences in HIV prevalence by socio-demographic variables and to assess differences among clinics.

## Conclusions

There is consistency in the HIV prevalence estimates from PMTCT programme and from antenatal HIV surveillance when antenatal HIV testing is routine standard of care. Our study underscores the validity and potential of the PMTCT programme estimates for monitoring trends in HIV/AIDS prevalence and to guide prevention and control efforts and are supported by previous studies. High PMTCT programme coverage, high rate of HIV testing in PMTCT programmes and good quality data are pre-requisites for successful transition from antenatal surveillance to PMTCT programme estimates whereby Addis Ababa seems to fulfil all. We recommend the Addis Ababa City Council Health Bureau and the respective sub-cities to use estimates from PMTCT programme reports for the purpose of HIV surveillance with continuous data quality monitoring using HMIS. Taking into account these results Ethiopia should continue to assess PMTCT data in order to replace ANC surveillance.

## Abbreviations

AIDS: Acquired immunodeficiency syndrome; DHS: Demographic and health survey; EIA: Enzyme Immunosorbent Assay; GHI: Global health initiative; HIV: Human immunodeficiency virus; PMTCT: Prevention of mother to child HIV transmission.

## Competing interests

The authors declare that they have no competing interest.

## Authors’ contributions

AHM prepared the study proposal, collected and analyzed the data, interpreted the findings and wrote the manuscript. OM was involved in developing the study proposal, supervising the data collection and reviewing the manuscript. SGH was involved in developing the study proposal and reviewing the manuscript. MMS was involved in supervising the data collection and reviewing the manuscript. KMM was involved in developing the study proposal and reviewing the manuscript. All authors read and approved the final manuscript.

## Authors’ information

AHM is a postdoctoral fellow at the Centre for International Health, University of Bergen Norway and is a corresponding author. MMS is an assistant professor at Addis Ababa University, Ethiopia. SGH, KMM and OM are professors at the Centre for International Health, University of Bergen, Norway.

## Pre-publication history

The pre-publication history for this paper can be accessed here:

http://www.biomedcentral.com/1471-2458/12/1113/prepub
